# A typhoid fever outbreak in a slum of South Dumdum municipality, West Bengal, India, 2007: Evidence for foodborne and waterborne transmission

**DOI:** 10.1186/1471-2458-9-115

**Published:** 2009-04-27

**Authors:** Rama Bhunia, Yvan Hutin, Ramachandran Ramakrishnan, Nishith Pal, Tapas Sen, Manoj Murhekar

**Affiliations:** 1 Field Epidemiology Training Programme, National Institute of Epidemiology (Indian Council of Medical Research), Chennai, India; 2 Department of Health and Family Welfare, West Bengal, India; 3 World Health Organization, India country office, New Delhi, India; 4 Department of Microbiology, School of Tropical Medicine, Kolkata, West Bengal, India

## Abstract

**Background:**

In April 2007, a slum of South Dumdum municipality, West Bengal reported an increase in fever cases. We investigated to identify the agent, the source and to propose recommendations.

**Methods:**

We defined a suspected case of typhoid fever as occurrence of fever for ≥ one week among residents of ward 1 of South Dumdum during February – May 2007. We searched for suspected cases in health care facilities and collected blood specimens. We described the outbreak by time, place and person. We compared probable cases (Widal positive >= 1:80) with neighbourhood-matched controls. We assessed the environment and collected water specimens.

**Results:**

We identified 103 suspected cases (Attack rate: 74/10,000, highest among 5–14 years old group, no deaths). Salmonella (enterica) Typhi was isolated from one of four blood specimens and 65 of 103 sera were >= 1:80 Widal positive. The outbreak started on 13 February, peaked twice during the last week of March and second week of April and lasted till 27 April. Suspected cases clustered around three public taps. Among 65 probable cases and 65 controls, eating milk products from a sweet shop (Matched odds ratio [MOR]: 6.2, 95% confidence interval [CI]: 2.4–16, population attributable fraction [PAF]: 53%) and drinking piped water (MOR: 7.3, 95% CI: 2.5–21, PAF-52%) were associated with illness. The sweet shop food handler suffered from typhoid in January. The pipelines of intermittent non-chlorinated water supply ran next to an open drain connected with sewerage system and water specimens showed faecal contamination.

**Conclusion:**

The investigation suggested that an initial foodborne outbreak of typhoid led to the contamination of the water supply resulting in a secondary, waterborne wave. We educated the food handler, repaired the pipelines and ensured chlorination of the water.

## Background

Typhoid fever is an acute febrile illness caused by Salmonella (enterica) Typhi [1,2]. The incubation period ranges from three days to one month [3]. Early symptoms include progressive onset of fever, headache, abdominal discomfort, loss of appetite, constipation followed by diarrhea, dry cough, malaise and rash along with relative bradycardia [2,3]. The case fatality ratio is 10% in the absence of treatment and less than 1% with antibiotics [1]. Human beings are the only natural reservoir. It is transmitted through ingestion of water or food that have been contaminated by feces or urine of patients and carriers [2,3]. Prevention is based on access to safe water and hygienic food handling practices [4]. In 2000, typhoid fever affected 21,650,974 million people worldwide and caused 216,510 deaths [5].

It is estimated that In Asia, the crude annual typhoid incidence rate was 274 per 100,000 persons in 2000 [5]. In India, typhoid incidence rates declined between 1974 and 1996 [5]. However, typhoid fever outbreaks continue to be reported in the country [6,7]. In 2004, the estimated incidence of typhoid was 104 per 100,000 populations in West Bengal [8].

In 2006, in the "North 24 Parganas" district of the Indian state of West Bengal, the incidence of typhoid fever was 124 per 100,000, three times more than in 2001. It was unclear whether this rise was real (increased incidence) or artefactual (increased reporting). In the Darjeeling district of the same state, improvement of reporting between 2000 and 2004 led to a 10 fold increase in five years [8]. On 12 April 2007, health authorities of the South Dumdum municipality in the North 24 Paraganas district (2001 population: 392,150, divided in 35 wards, [9]) noted and reported an increase in the number of fever cases reported passively from a slum. This municipality is in the suburban area of Kolkata, the Capital of West Bengal. We investigated this cluster to identify the causative agent, identify the source, control the outbreak and propose recommendations to prevent future occurrences.

## Methods

### Descriptive epidemiology

We compiled monthly municipal surveillance data of fever from January 2004 to May 2007. We reviewed information regarding any recent change in the case definition, surveillance or population size. We defined a suspected case of typhoid fever as occurrence of fever (Temperature > 38°C) for one week or more in a resident of ward-1, South Dumdum municipality (2007 projected population: 13,920) between February and May 2007 [10] (Fever of a one week or more duration is the syndromic case definition for typhoid in the national Indian surveillance system). We searched for suspected cases in health camps and health care facilities. We interviewed each of the suspected case-patient and reviewed medical records to collect information regarding demographic characteristics, signs and symptoms, number of household members present and affected, food habits, laboratory investigations and outcome. We described the outbreak over time. We calculated the attack rate by age and sex. We constructed a spot map. We collected information from a random sample of suspected case-patients and those with unusual characteristics using open, unstructured interviews to generate hypotheses about potential sources of infection.

### Laboratory investigations

We abstracted medical record of suspected case-patients for evidence of Widal test results. In addition, we collected four acute phase blood specimens from a random sample of suspected case patients who had not taken antibiotics. We sent those to the School of Tropical Medicine in Kolkata for blood culture.

### Case control study

We conducted a matched case control study during the first 10 days of May 2007. We defined a probable case of typhoid fever as a suspected case with a Widal titre of >= 1:80 (The threshold used in Kolkotta for the diagnosis of typhoid since the background titre is 1:40, Dr Pal, Kolkota School of Tropical Medicine, Personal Communication). We compared each probable case-patient with a control subject selected from the next-door neighbours without history of fever or typhoid. We designed a structured questionnaire, translated it into Bengali -the local language-, pre-tested it (with unaffected community members not included as control-subjects) and back translated it again into English. We trained field workers in data collection. We collected information regarding demographic characteristics, food handling practices, use of drinking water, and sanitation practices in the 14 days prior to onset (probable cases) or recruitment (controls). We calculated matched odds ratio (MOR) for discordant pairs and 95% confidence interval (CI) using Epi Info version 3.3.2. We calculated the attributable fraction in the population (PAF) for the exposures for which we suspected causality using the formula of the proportion of cases exposed multiplied by the attributable fraction among exposed (odds ratio-1/odds ratio). We protected confidentiality of participants through codes and obtained oral consent before interviews. The investigation was exempted from ethical committee clearance since it was part of the state level public health response to the outbreak.

### Environmental investigations

As hypothesis-generating interviews among suspected case-patients and the geographical distribution pointed to a sweet shop, we interviewed the shop owner, its workers and reviewed their hygienic practices. We conducted open interviews with suspected case patients, health workers and local leaders to collect information regarding the local water supply and sanitation. We visited ward 1 to assess the sanitary situation and collected water specimens from tap water and tube well water for bacteriological analysis.

## Results

### Descriptive epidemiology

**Figure 1:**
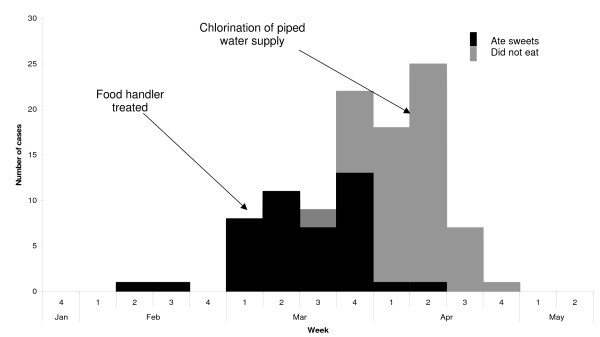
**Suspected cases of typhoid (N = 103) by date of onset in ward 1, South Dumdum municipality, "North 24 Parganas" district, West Bengal, India, January–May 2007**.

**Figure 2:**
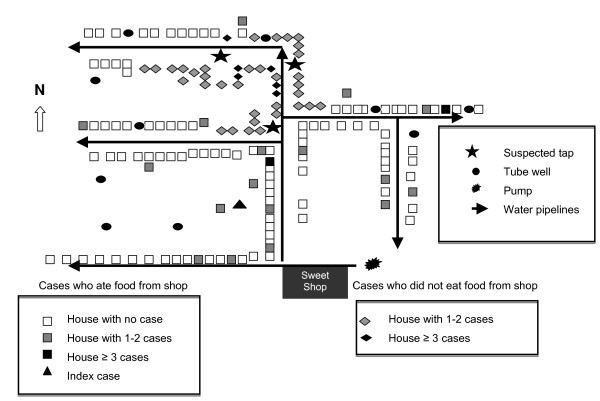
**Distribution of possible cases (Fever of at least one-week duration) in the affected area of ward 1, South Dumdum municipality, "North 24 Parganas" district, West Bengal, India, February–May 2007 (N = 103)**.

The average reported monthly rate of fever of >= one-week duration ranged between 4 and 8 per 10,000 population during 2004 and 2007 in the urban health sub-centre of ward 1. From February to April 2007, we identified 103 suspected cases (Incidence: 74 per 10,000). Clinical symptoms included malaise, loss of appetite, headache, cough, constipation, diarrhea and rash (Table 1). The median age of suspected case-patients was 13 years (Range 2–77), six percent of suspected case-patients had been hospitalized and there were no deaths. The attack rate among the 5 to 14 years of age was 151 per 10,000, twice higher than among other age groups (Table 2). Trawling questionnaires yielded frequent exposures to a sweet shop in ward 1 among suspected case-patients. The outbreak started on 13 February, had two peaks on during the last week of March and second week of April and ended on 27 April (Figure 1). Suspected cases clustered around three public taps located about 50 metres away from the sweet shop (Figure 2). Suspected case-patients who reported consumption of food from the sweet shop had an earlier date of onset than others (Figure 1). Based on (1) the distribution of suspected cases over time, (2) the trawling questionnaires that pointed to a sweet shop and (3) the geographical distribution of suspected cases around a specific source of water supply, we generated the hypothesis that two sources i.e. food and tap water could be the source of the outbreak.

**Table 1:** Signs and symptoms of cases of fever of at least one week duration by timing of onset (N = 103), ward 1 of South Dumdum Municipality, "North 24 Parganas" district, West Bengal, India, February–May 2007

	Early ^1^ (n = 43)		Late ^2^ (n = 60)		Total	
	
Characteristics	#	%	#	%	Number	Proportion (%)
Malaise	43	100	60	100	103	100
Headache	39	91	55	92	94	91
Anorexia	40	93	53	88	93	90
Cough	7	16	10	17	17	17
Constipation	4	9	5	8	9	9
Diarrhea	3	7	5	8	8	8
Hospitalized	2	5	4	7	6	6
Rash over trunk	1	2	0	0	1	1
Death	0	0	0	0	0	0

^1^Before 1 April 2007

^2^1 April 2007 and after

**Table 2:** Attack rates of cases of fever of at least one week duration by age and sex, ward 1 of South Dumdum municipality, "North 24 Parganas" district, West Bengal, India, February–May 2007

**Demographic characteristics**		**Number of cases**	**Population **^1^	**Attack rate per 10,000**
**Age**	**0–4**	9	1,322	68
	**5–14**	50	3,305	151
	**15–24**	23	2,553	90
	**25–34**	16	2,309	69
	**35–44**	3	1,843	16
	**45+**	2	2,588	08

**Sex**	**Male**	45	7,199	63
	**Female**	58	6,721	86

**Total**		103	13,920	74

^1^2007 projection of the 2001 census

### Laboratory investigations

According to medical records, 65 of the 103 suspected case-patients sera were positive for the Widal test (>= 1:80), seven were negative and 31 had not been tested. One out of four blood specimens grew Salmonella (enterica) Typhi.

### Case control study

We recruited 65 probable cases (Median age: 14 years) and 65 controls (Median age: 18 years, p = 0.11) for the case control study (Table 3). Probable cases were more likely than controls to have a monthly household income under 1,500 rupees, to take milk products from the nearby sweet shop (Matched odds ratio [MOR]; 6.2, 95% confidence interval [CI]: 2.4–16), to drink only piped water (MOR 7.3, 95% CI: 2.5–21) and to have more than four household members. We did not ask questions regarding consumption of specific food items from the sweet shop. Compared to controls, probable cases were less likely to wash hands with soap after defecation or urination, to use of spoon to serve food, to purify drinking water, to store water in narrow containers and to cover water containers (Table 3). Population attributable fraction suggested that 53% and 52% of probable cases might have been attributable to the sweet shop and the piped water, respectively. Both risk factors were independent risk factors in an unmatched stratified analysis (Data not shown).

**Table 3:** Matched sets according to the exposure status of the suspected typhoid cases (n = 65) and controls (n = 65), South Dumdum municipality, "North 24 Parganas" District, West Bengal, India, February 2007

		**Number of case control pairs according to exposure status**				
			
**Characteristics**		**Concordant**		**Discordant**		**Matched odds ratio** **(95% CI)**^1^
				
		**Case exposed**	**Case unexposed**	**Case exposed**	**Case unexposed**	
**Demographic status**	**Sex**	12	25	13	15	0.87 (0.41–1.8)
	**Education > secondary**	46	1	4	14	0.28 (0.09–0.87)
	**Household > 4 members**	8	26	25	6	4.2 (1.7–11.1)
	**Monthly family income < 1500 INR**	1	50	12	2	6.0 (1.3–26.8)
	**Anyone ill in neighborhood**	11	19	25	10	2.5 (1.2–5.2)

**Food habits**	**Eating outside**	30	2	20	13	1.5 (0.8–3.1)
	**Food from sweet shop **^2^	10	19	31	5	6.2 (2.4–16)
	**Food from shop S**	1	48	9	7	1.3 (0.48–3.5)
	**Food from shop G**	7	30	9	19	0.47 (0.20–1.0)
	**Sweets**	11	19	19	16	1.2 (0.60–2.3)
	**Curd**	6	31	17	11	1.5 (0.72–3.3)
	**Paratha**	1	42	15	7	2.1 (0.87–5.3)
	**Street ghugni**	0	43	14	8	1.8 (0.73–4.2)
	**Street pickle**	0	58	3	4	0.75 (0.17–3.4)
	**Street panipuri**	8	30	14	13	1.1 (0.50–2.3)
	**Ice cream**	7	28	18	12	1.5 (0.72–3.1)
	**Use of spoon to serve food**	23	3	1	38	0.03 (0–0.2)

**Drinking water**	**Piped water only**	10	22	29	4	7.3 (2.5–21)
	**Tube well water only**	34	11	4	16	0.25 (0.08–0.75)
	**Purification**	3	36	8	18	0.44 (0.19–1.0)
	**Covered container**	44	1	4	16	0.25 (0.08–0.75)
	**Narrow mouth container**	5	29	8	23	0.35 (0.15–0.76)

**Hygienic practices**	**Soap hand wash before food**	41	1	15	8	1.9 (0.80–4.4)
	**Soap hand wash after defecation**	38	1	6	20	0.30 (0.12–0.75)
	**Soap hand after urination**	9	16	3	37	0.08 (0.03–0.26)

^1^Confidence interval

^2^Milk products

### Environmental investigations

The sweet shop sold yogurt and sweets. The food handler who prepared sweets did not wash hands to serve customers. He suffered from typhoid fever (confirmed by an increase of titre in two paired Widal tests) during the last week of January 2007, prior to this outbreak. He served food during his illness and sought treatment at the end of February, which resulted in a resolution of his symptoms. None of his stools could be cultured.

Ward 1 had two types of drinking water sources: tube well water and pipeline water. The tube well located in the area where the suspected cases clustered, had a concrete platform and its water was free from coliforms. One direct ground pump supplied water through pipelines intermittently, three times a day. This water source was not chlorinated. The water pipelines ran closely to an open drainage system that was the only form of sanitation in the area. Water specimens collected from three of four taps selected at random among those supplied by the pipelines were positive for coliforms (range 15–32/100 ml). The water stored in wide mouthed containers in affected households, had higher coliform count (41/100 ml) than the fresh tap water specimens. The population used common sanitary latrines or latrines that connected directly to the open drainage.

## Discussion

A number of elements suggested that part of this outbreak was foodborne. First, there was an association between eating food from the sweet shop and illness, and that exposure explained about half of the cases. Second, there was an infected source patient working in the sweet shop who engaged in unsafe food handling practices three weeks before the occurrence of the outbreak. The distribution of the suspected case-patients with exposure to the food from the shop over time suggested that this exposure explained the first part of the outbreak. Among typhoid outbreaks, food borne episodes are common in several countries [11-18]. Following contamination by an infected patient, milk-based sweets would have constituted good growth media for the bacterium.

The second part of the outbreak suggested waterborne transmission. Several studies reported typhoid fever outbreak as waterborne [19-22]. A number of elements suggested that the piped water accounted for a number of cases during this outbreak. First, suspected cases clustered in a location that was consistent with a contamination of the piped water. Second, there was an association between exclusive consumption of piped water and illness that explained about half of the cases. Third, analysis of the water specimen supported the hypothesis of contamination. The distribution of the suspected cases over time suggested that the piped water supply explained the second part of the outbreak. While there was no direct evidence to explain the sequence between the two parts of the outbreak, the environmental assessment suggested a possible scenario. The patients affected during the first phase of the outbreak excreted the pathogen in the environment. In a context of a poor sanitation system where water pipelines with negative pressure on account of intermittent water supply, ran closely to the open drainage system, the contaminated sewage may have been sucked in the piped water distribution system. Once the bacteria were transmitted in the community, unsafe water handling practices may also have led to a number of cases. Stored water was significantly associated with the disease when in uncovered or wide mouthed container and stored water was contaminated with fecal coliform. Narrow mouthed containers have been shown to prevent secondary transmission of typhoid [21,22]. Water storage in a covered container (that helps prevent contaminations) may be associated with a lower risk of disease [21]. Other findings of our case control study were consistent with reported risk factors for typhoid fever, including high attack rates among children [23-25], large households [25] and families living in poor sanitation environment [26-29].

This study had one main limitation. We had only one positive blood culture for Salmonella (enterica) Typhi. Thus, we cannot exclude the possibility that a number of the 103 suspected cases had other forms of infections and we may have overestimated the size the outbreak. In the case control study, we included only probable cases that had a Widal test >= 1;80. The use of a single Widal agglutination test for the diagnosis of typhoid is controversial, particularly when the reagents are not standards and when there are no data on the local sensitivity and specificity for a given dilution [30]. However, in the context of India, it is still used as (1) a 1:80 titre provides acceptable sensitivity and specificity in the absence of other diagnostic options and (2) the positive predictive value is higher in a context of high frequency of the disease [31]. The clinical symptoms were compatible with the diagnosis of typhoid fever and did not change during the two phases of the outbreak. Furthermore, if we included patients with other infections in the case control study, this would have led to non-differential misclassification that would not prevent our ability to conclude with respect to the sources of infection identified.

## Conclusion

This probable typhoid fever outbreak presented with a unique sequence with a first phase associated with eating food from a sweet shop and a second phase secondary to a contamination of the piped water supply. Unsafe water handling practices might also have contributed to the spread. On the basis of these conclusions, we formulated a number of recommendations. First, we educated the food handler of the shop regarding use of soap before sweet preparation and use of spoon to serve food. Second, we recommended daily chlorination of the piped water. We ensured the implementation of this recommendation through several meetings with the local authorities and engineers. After chlorination of the piped water supply, the outbreak came to an end. Third, we conducted health education with the community regarding safe water handling practices. For the long-term control of typhoid, both food safety and water safety will be needed, and adequate sanitation should be achieved to prevent the type of environmental contaminations that triggered a waterborne outbreak after a foodborne one. Epidemiologists investigating typhoid and other outbreaks transmitted through the fecal-oral route must always keep in mind the possibility of combined mixed modes of transmission during outbreaks. Finally, efforts must be made to evaluate newer innovative strategies for the diagnosis of typhoid fever in India so that the common use of the Widal test may be replaced with a better option, possibly with the use of rapid tests (e.g., Typhi-Dot) that have been proposed in the context of the new national surveillance programme.

## Competing interests

The authors declare that they have no competing interests.

## Authors' contributions

RB concept, design, acquisition of data, analysis, interpretation and writing of manuscript. YH concept, analysis and critical drafting of manuscript. RR analysis and interpretation. NP laboratory analysis and critical revision of manuscript. TS interpretation and drafting the manuscript. MM analysis, interpretation and writing of manuscript. All authors read and approved the final manuscript.

## Pre-publication history

The pre-publication history for this paper can be accessed here:


